# Natural variation in DNA methylation in ribosomal RNA genes of *Arabidopsis thaliana*

**DOI:** 10.1186/1471-2229-8-92

**Published:** 2008-09-10

**Authors:** Hye Ryun Woo, Eric J Richards

**Affiliations:** 1 Department of Biology, Washington University, One Brookings Drive, St. Louis, MO 63130, USA

## Abstract

**Background:**

DNA methylation is an important biochemical mark that silences repetitive sequences, such as transposons, and reinforces epigenetic gene expression states. An important class of repetitive genes under epigenetic control in eukaryotic genomes encodes ribosomal RNA (rRNA) transcripts. The ribosomal genes coding for the 45S rRNA precursor of the three largest eukaryotic ribosomal RNAs (18S, 5.8S, and 25–28S) are found in nucleolus organizer regions (NORs), comprised of hundreds to thousands of repeats, only some of which are expressed in any given cell. An epigenetic switch, mediated by DNA methylation and histone modification, turns rRNA genes on and off. However, little is known about the mechanisms that specify and maintain the patterns of NOR DNA methylation.

**Results:**

Here, we explored the extent of naturally-occurring variation in NOR DNA methylation among accessions of the flowering plant *Arabidopsis thaliana*. DNA methylation in coding regions of rRNA genes was positively correlated with copy number of 45S rRNA gene and DNA methylation in the intergenic spacer regions. We investigated the inheritance of NOR DNA methylation patterns in natural accessions with hypomethylated NORs in inter-strain crosses and defined three different categories of inheritance in F1 hybrids. In addition, subsequent analysis of F2 segregation for NOR DNA methylation patterns uncovered different patterns of inheritance. We also revealed that NOR DNA methylation in the Arabidopsis accession Bor-4 is influenced by the *vim1-1 *(*variant in methylation 1-1*) mutation, but the primary effect is specified by the NORs themselves.

**Conclusion:**

Our results indicate that the NORs themselves are the most significant determinants of natural variation in NOR DNA methylation. However, the inheritance of NOR DNA methylation suggests the operation of a diverse set of mechanisms, including inheritance of parental methylation patterns, reconfiguration of parental NOR DNA methylation, and the involvement of *trans*-acting modifiers.

## Background

DNA methylation is an important mechanism for establishing stable heritable epigenetic marks that modify the information content of the underlying genetic sequence [1]. In plants and vertebrates, DNA methylation is important for gene regulation, genomic imprinting, heterochromatin assembly, and protection of the genome against migrating transposable elements [2,3]. Both forward and reverse genetic approaches have identified important components of DNA methylation systems in eukaryotes, including cytosine-DNA-methyltransferases (DNMTs) and chromatin modification enzymes [2,4,5]. In addition to conventional genetic approaches, research based on the analysis of natural genetic variation has increased dramatically in recent years [6-9]. In the flowering plant, *Arabidopsis thaliana*, this approach has relied on an expanding catalog of natural accessions collected from around the world [10,11]. The analysis of genetic variation among Arabidopsis accessions has identified components important for various aspects of the plant's biology, including the circadian clock [12], flowering time [13-15], pathogen resistance [16], and uncovered natural variation for drought responses [17] and freezing tolerance [18]. Natural accessions also provide a potential source of natural epigenetic variation. For example, meiotically transmissible epialleles of a novel non-LTR retroposon family (*Sadhu*) were identified through mining of naturally-occurring epigenetic alleles in Arabidopsis [19]. Exploring Arabidopsis accessions also revealed that DNA methylation within the coding sequences of genes is highly polymorphic among Arabidopsis natural accessions and that these polymorphisms in genic methylation behave as heritable variation in inter-strain crosses [20].

Nucleolus organizer regions (NORs) are composed of several hundred copies of tandemly-arrayed ribosomal RNA (rRNA) genes encoding the 45S precursor transcript for the three largest ribosomal RNAs (18S, 5.8S and 25S-28S rRNAs (the size is species-dependent)). In Arabidopsis, rRNA genes are clustered in two NORs, *NOR2 *and *NOR4*, each of which is composed of the hundreds of rRNA genes arranged head-to-tail in an uninterrupted array [21]. rRNA transcription is a massive energy-consuming process, necessitating tight and economic regulatory mechanisms for rRNA transcription [22,23]. One of the mechanisms that regulate rRNA transcription is the control of the number of rRNA genes in the on or off state. Several lines of evidence implicate an epigenetic switch in rRNA gene promoter DNA methylation and histone modification [24,25]. However, our understanding of the mechanisms that specify and maintain the patterns of DNA methylation in NORs remain incomplete.

In this study, we explored the extent of variation in DNA methylation in Arabidopsis natural accessions by focusing on the methylation of the major rRNA gene repeats at the two NORs. We extended our previous studies [26] on natural variation of DNA methylation in the coding regions (CR) of rRNA genes and the correlation between CR DNA methylation and rRNA gene copy number. Furthermore, we investigated DNA methylation of the intergenic sequences (IGS) in natural accessions. Our results indicate that there is a positive but imperfect correlation between CR and IGS DNA methylation, suggesting the existence of independent as well as common regulatory mechanisms controlling DNA methylation in these two different regions of the 45S rRNA gene repeats. We also defined new modes of the inheritance of NOR DNA methylation involving the reconfiguration of parental NOR DNA methylation in hybrids and the action of *trans*-acting modifiers.

## Results

### Natural variation in DNA methylation of major rRNA gene repeats in Arabidopsis

**Figure 1:**
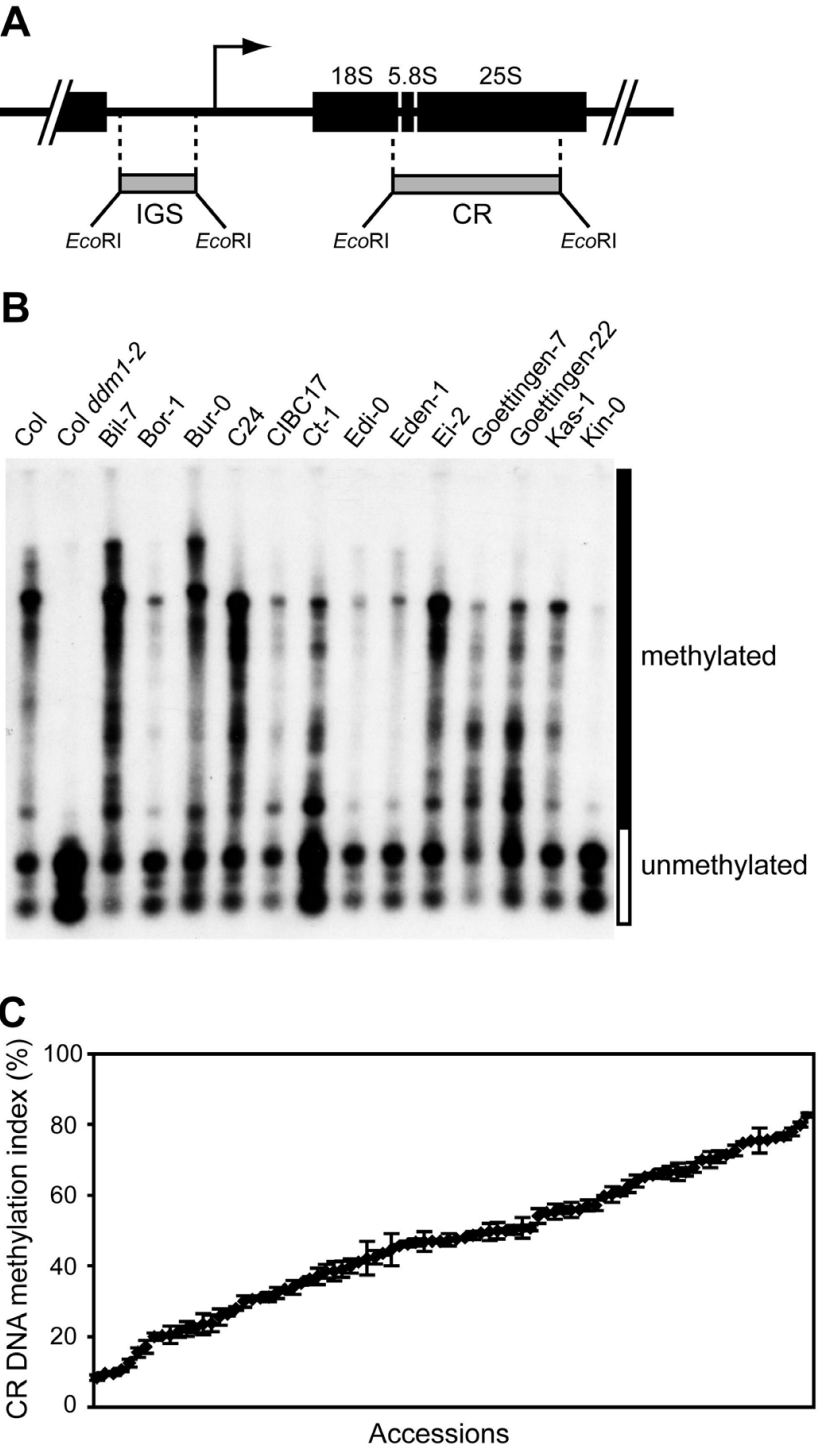
DNA methylation in 45S rRNA gene repeats among Arabidopsis natural accessions. (A) A 10-kb rRNA gene repeat unit encoding the 18S, 5.8S, and 25S rRNAs. A bent arrow shows the location of the transcription start site, and the two hybridization probes (IGS and CR) are indicated below the monomer repeat unit as gray bars. (B) A representative genomic DNA blot analysis of Arabidopsis natural accessions using the CR probe. *Hpa*II-digested genomic DNA was size fractionated by gel electrophoresis and transferred to a nylon membrane. The membrane was hybridized with radiolabeled CR probe corresponding to the 5.8S and 25S rRNA coding regions (see the map above). The hybridization signal indicates the extent of DNA methylation in the rRNA gene clusters; unmethylated fragments are cleaved by *Hpa*II to small fragments (open box), whereas methylated genomic fragments remain uncleaved, or partially cleaved, and migrate as larger fragments (solid box). (C) Variation in CR DNA methylation among 88 Arabidopsis natural accessions. CR DNA methylation index (%) was calculated by measuring the percentage of hybridization signal in each lane that corresponded to restriction fragments >1 kb. Means and standard errors were calculated from at least three independent experiments.

Our previous study of a limited number of Arabidopsis natural accessions uncovered significant variation in DNA methylation of coding regions of 45S rRNA gene repeats [26]. Here, we examined a larger collection of Arabidopsis accessions to investigate natural variation in NOR DNA methylation in more detail. The extent of rRNA gene methylation was evaluated in 88 accessions of *A. thaliana *collected by Nordborg *et al*. [27] using DNA gel blot analysis with the methylation-sensitive restriction endonuclease, *Hpa*II (5'-CCGG-3'), which is inhibited by either 5mCpG or 5mCpHpG (Fig. 1). A hybridization probe corresponding to the 5.8S and 25S rRNA coding sequences (CR) should detect only <1 kb fragments following complete cleavage of the 70 *Hpa*II sites within the entire 10 kb rRNA gene (Fig. 1A). Figure 1B and Additional File 1 show representative genomic DNA blot analyses demonstrating that the extent and pattern of DNA methylation in the CR of the 45S rRNA gene repeats varies greatly among Arabidopsis natural accessions. The examined *Hpa*II sites were largely methylated in a standard laboratory strain Columbia (Col), and these *Hpa*II sites were unmethylated in the Col *ddm1-2 *(*decrease in DNA methylation 1–2*) mutant [28]. Based on the distribution of CR hybridization signal in the Col *ddm1-2 *mutant, we calculated CR DNA methylation indices by measuring the percentage of hybridization signal in each lane that corresponded to restriction fragments larger than 1 kb. Figure 1C and Additional File 2 summarize CR DNA methylation indices in the different Arabidopsis natural accessions examined, which varied widely over a range from 8.1% in Bay-0 to 82.5% in Bil-7. Our broader study of CR DNA methylation among 88 accessions further demonstrates that considerable natural variation exists in rRNA gene methylation in Arabidopsis.

Even in natural accessions with similar CR DNA methylation indices, DNA methylation appears to be differentially distributed along the rRNA gene repeat. For instance, Goettingen-7 and Col had comparable CR DNA methylation indices (69.9% and 69.8%, respectively) [see Additional File 2], yet Goettingen-7 displayed an enriched hybridization signal from restriction fragments of intermediate size, while most of the signal in Col corresponded to unmethylated or highly methylated restriction fragments (Fig. 1B). These differences cannot be easily explained by nucleotide sequence polymorphisms among the accessions because rRNA coding sequences are highly conserved within plant species [29,30]. Moreover, in light of this conservation, the distinct patterns of CR DNA methylation suggest that the variation in DNA methylation has a significant epigenetic component.

### Detailed DNA methylation patterns for natural accessions with hypermethylated or hypomethylated NORs

We were concerned that our reliance on *Hpa*II digestion in the DNA blot analysis described above might create a bias against heavily methylated rRNA gene repeats due to the limited mobility of larger genomic DNA fragments in agarose gels and the potential for uneven hybridization. To verify our *Hpa*II digestion results, we performed DNA gel blot analyses after digesting genomic DNA with *Hpa*II and *Eco*RI. As two *Eco*RI sites are located at each end of the CR hybridization probe (Fig. 1A), simultaneous digestion of *Hpa*II+*Eco*RI will give information on DNA methylation within the 3.7 kb coding region of rRNA gene repeats (containing 32 *Hpa*II sites) without interference from flanking regions and the concern about gel blot hybridization artifacts. For this analysis, we chose 29 natural accessions including 10 strains with high CR DNA methylation indices (>65%) and 16 strains with low CR DNA methylation indices (<30%). Additional File 3A shows a representative genomic DNA blot analysis using *Hpa*II+*Eco*RI digestion. CR DNA methylation indices based on *Hpa*II and *Hpa*II+*Eco*RI demonstrated a strong positive correlation (linear correlation coefficient, R = 0.99) [see Additional File 3B]. This finding supports the use of *Hpa*II digestion as an accurate representation of NOR DNA methylation.

To quantify CR DNA methylation in more detail we measured the relative hybridization signal intensity from unmethylated fragments (fraction a), a single band corresponding to the undigested 3.7 kb fragments (fraction c), and fragments migrating at positions intermediate (fraction b) between the fully methylated and unmethylated fragments. Most examined hypermethylated (>65% methylation index) accessions displayed a common pattern of CR DNA methylation: the weakest hybridization signal originated from the unmethylated fragments (fraction a), the strongest signal from the fully methylated fragments (fraction c), and a moderate signal from partially methylated fragments (fraction b) [see Additional File 4A]. However, two natural accessions with high CR DNA methylation indices demonstrated distinct CR DNA methylation patterns relative to the common pattern observed in other hypermethylated accessions. Ler-1 showed a similar signal intensity from unmethylated fragments (fraction a, 27%) and partially methylated fragments (fraction b, 28%). In contrast, C24 showed a relatively lower signal from the fully methylated fragment (fraction c, 39%) and a similar signal intensity from fractions b (38%) and c [see Additional File 4B].

In the case of strains with hypomethylated rRNA genes, all 16 accessions (<30% methylation index) displayed the most intense signals from unmethylated fragments (fraction a, 71–94%) [see Additional File 4C–E]. Four natural accessions with CR DNA methylation indices of ~30% showed two qualitatively different DNA methylation patterns. In one group comprised of accessions KZ9 and Spr1-6, the relative hybridization intensity of fractions b and c were similar, while the second group, containing Ts-5 and Zdr-6, showed a relatively high hybridization signal in fraction b and almost no signal from fraction c [see Additional File 4D, E]. These results indicate that there is significant natural variation not only in total CR DNA methylation but also in qualitative rRNA gene methylation patterns among Arabidopsis natural accessions.

### rRNA gene copy number contributes to CR DNA methylation in Arabidopsis natural accessions

**Figure 2:**
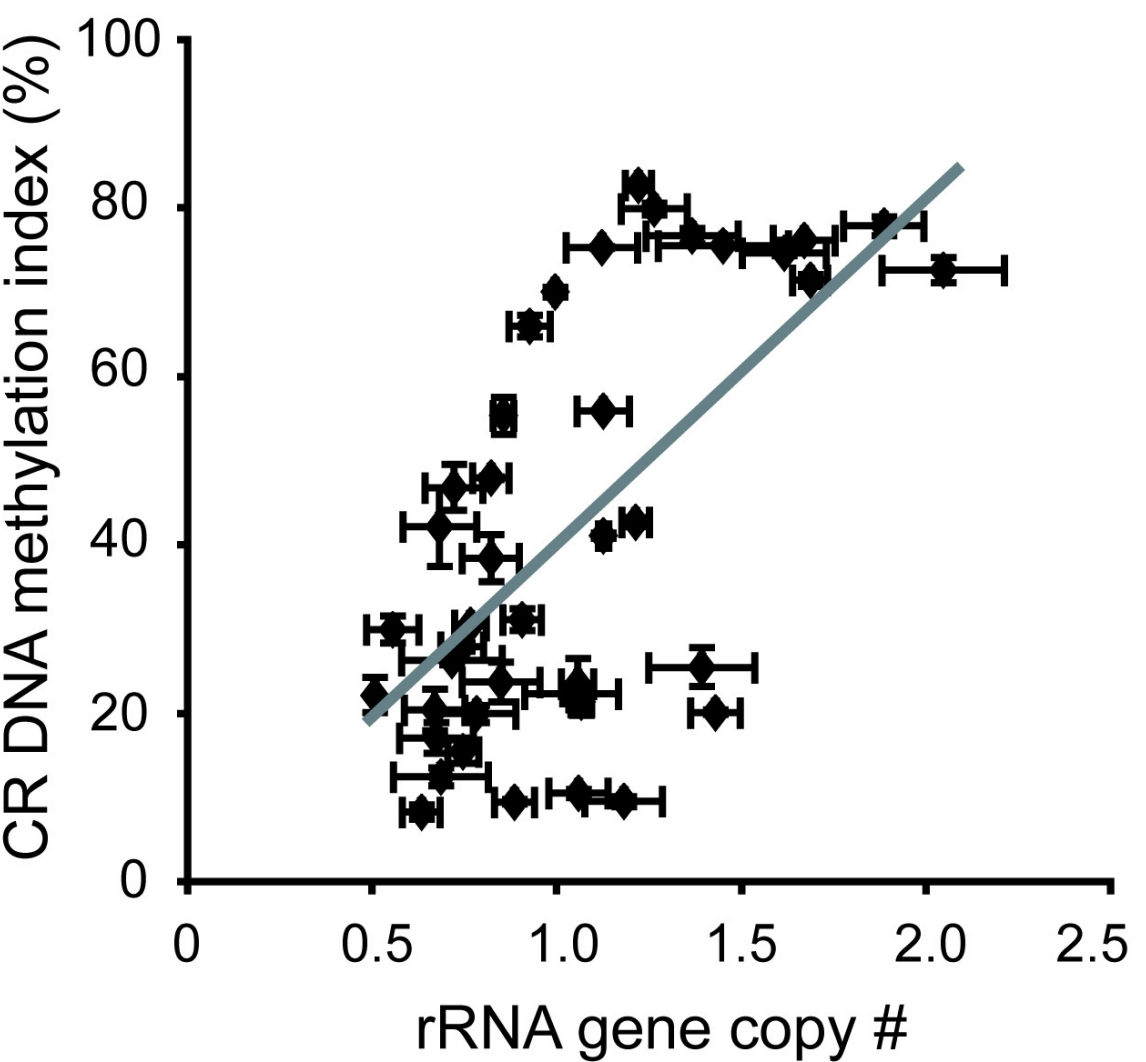
Positive correlation between rRNA gene copy number and CR DNA methylation. CR DNA methylation indices were plotted against normalized rRNA gene copy numbers in 41 Arabidopsis natural accessions. Means and standard errors were calculated from at least three independent experiments. The gray line is a linear regression of the data (linear regression coefficient, R = 0.63).

To study the basis of the observed variation in CR DNA methylation among natural accessions we investigated the relationship between rRNA gene copy number and CR DNA methylation in 41 natural strains from the collection of 88 accessions described in the previous section. Among the 41 accessions, the relative rRNA gene copy number compared to that of the standard reference strain Col ranged from 0.51× in Sq-1 to 2.05× in KZ1 [see Additional File 2]. Consistent with our previous findings, which were based on a smaller collection of accessions [26], we found that the copy number of rRNA genes was positively correlated with CR DNA methylation in this larger survey (Fig. 2). The effect of the relative copy number of rRNA genes on CR DNA methylation was assessed by linear regression and a significant correlation was observed between the two parameters (linear correlation coefficient, R = 0.63). All natural accessions with CR DNA methylation indices of >70% had rRNA gene copy numbers ≥ 1.13× that of the Col strain and all accessions with CR DNA methylation indices of <16% had rRNA gene copy numbers 1.19× or lower compared to the Col strain. Nonetheless, several natural accessions with comparable levels of CR DNA methylation had markedly different rRNA gene copy number. For example, strains CS22569 and Zdr-6 shared CR DNA methylation indices of ~20%, but CS22569 had one-half the number of rRNA genes present in Zdr-6 (0.67× Col for CS22569 versus 1.43× Col for Zdr-6). Similar discrepancies between rRNA gene copy number and CR DNA methylation were also found among accessions with high CR DNA methylation indices (for example, compare C24 and KZ1). Overall our results suggest that rRNA gene copy number is one of the main determinants for CR DNA methylation, but other factors must also contribute to CR DNA methylation.

### Natural variation of DNA methylation in the IGS of 45S rRNA genes

**Figure 3:**
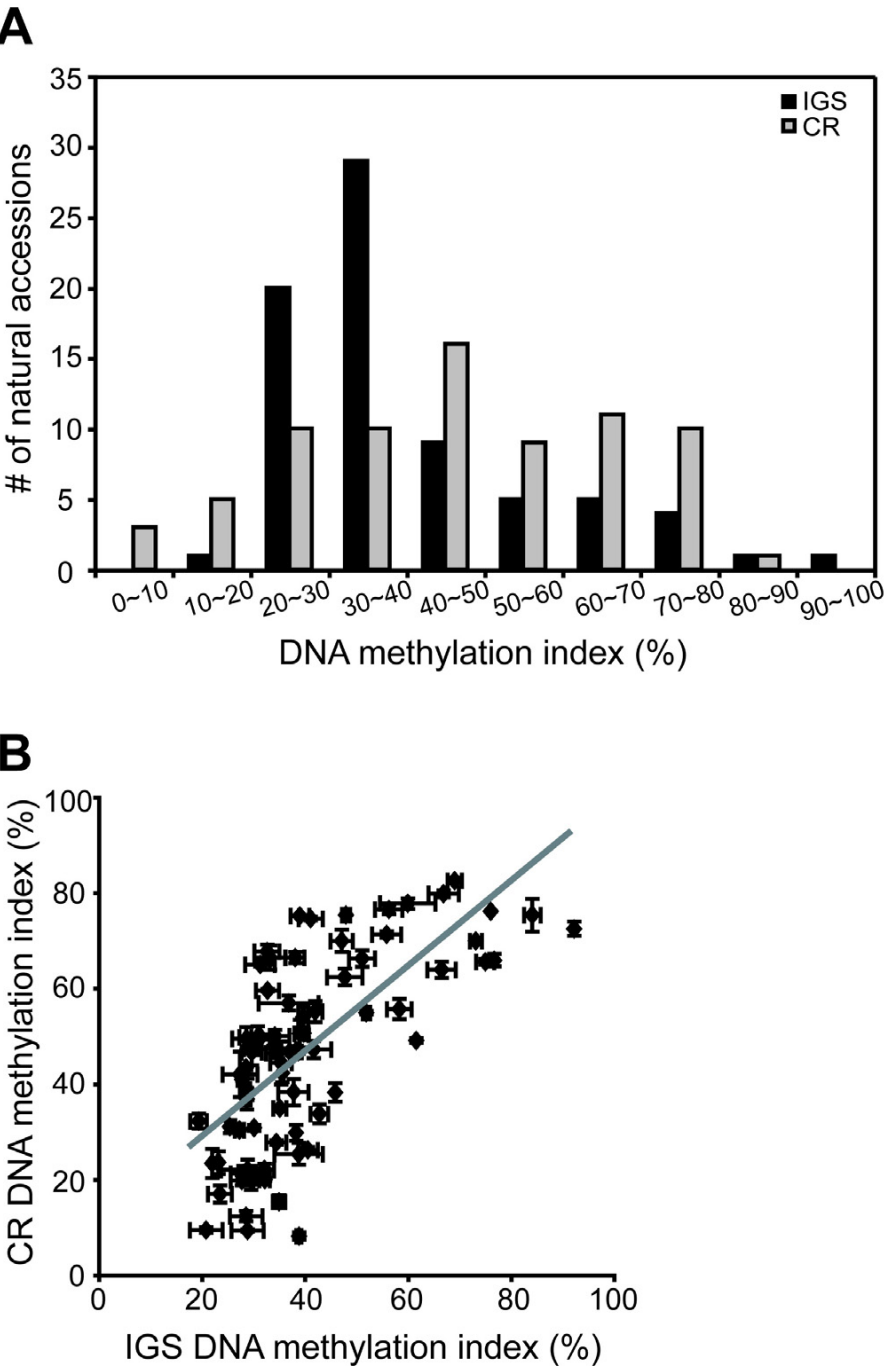
IGS DNA methylation in 45S rRNA gene repeats among Arabidopsis natural accessions. (A) Distributions of relative CR and IGS DNA methylation in 75 natural accessions. The frequency histograms show the range and distribution of CR and IGS DNA methylation in Arabidopsis natural accessions. (B) A correlation between CR DNA methylation and IGS DNA methylation. Means and standard errors were calculated from at least three independent experiments. The gray line is a linear regression of the data (linear regression coefficient, R = 0.68).

Tandemly-arrayed rRNA coding sequences are separated from one another by an intergenic spacer containing repetitive elements as well as the rRNA gene promoter. To investigate whether IGS regions have distinct DNA methylation patterns from the CR regions in rRNA genes, we used *Hpa*II digestion to measure the DNA methylation content in the IGS (containing 16 *Hpa*II sites) and compared the distribution of DNA methylation indices in the CR and IGS regions. The number of natural accessions with significantly higher CR DNA methylation relative to IGS DNA methylation was almost three times more than the number of accessions with higher IGS DNA methylation than CR DNA methylation [see Additional File 2]. Moreover, CR DNA methylation indices were broadly distributed, with a relatively equal distribution of accessions over the 20 to 80% range. In contrast, IGS DNA methylation indices for the majority of accessions (49 out of 75 accessions examined) fell within a narrow 20 to 40% window (Fig. 3A).

Figure 3B shows a positive correlation between DNA methylation in the CR and IGS, suggesting that common mechanisms regulate DNA methylation in the CR and IGS of the 45S rRNA gene repeats. However, the divergence of DNA methylation in the two regions of the rRNA genes in some accessions indicates that the relationship between DNA methylation in these two regions is more complex. For example, some natural accessions (Bay-0, C24, CS22568, Goettingen-22, Lov-1, and Ts-1) displayed significant differences (>30%) between DNA methylation in the CR and IGS [see Additional File 2]. Viewed from another perspective, there are natural accessions which had similar IGS DNA methylation but extremely different CR DNA methylation [see Additional File 2]. For instance, Bay-0 and C24 had IGS DNA methylation indices of 38.9% and 39.1%, but CR DNA methylation indices of 8.1% and 75.1%, respectively. KZ1 and Lov-1 displayed the opposite pattern; KZ1 and Lov-1 had CR DNA methylation indices of 72.4% and 74.5%, but had IGS DNA methylation indices of 92.3% and 41.2%, respectively. The patterns of CR and IGS DNA methylation among Arabidopsis natural accessions suggest that IGS and CR DNA methylation might be coordinated in a general sense, but there also are independent regulatory mechanisms that affect DNA methylation in these two different regions of the 45S rRNA gene repeats.

### Genetic analyses of hybrids between Col and accessions with hypomethylated NORs

To investigate how NOR DNA methylation patterns are inherited, we examined CR DNA methylation levels in F1 individuals generated by crosses between the standard lab strain Col and 16 low CR DNA methylation accessions. The accessions were categorized into three groups based on the CR DNA methylation patterns in F1 hybrids. 10 out of 16 low CR DNA methylation accessions belonged to group I, in which the average CR DNA methylation indices in F1 individuals were intermediate between the two parental values (Table 1). Group II included two natural accessions that resulted in F1 hybrids with Col that exhibited lower CR DNA methylation than the weighted mid-parental value. In contrast, group III contained four accessions characterized by F1 hybrids that exhibited significantly higher CR DNA methylation than the expected weighted mid-parental value. The results observed for crosses with group I strains are consistent with the faithful inheritance of parental methylation patterns that we have previously documented [31]. The unexpected results from the group II and III crosses suggest that reconfiguration of parental NOR DNA methylation occurred in the hybrids.

**Table 1:** Inheritance of NOR DNA methylation in F1 hybrids

Group	Accessions	CR DNA methylation index (%)	Relative rRNA gene copy number	CR DNA methylation index in F1 hybrids with Col	
				
				Expected (%)	Experimental (%)^a^
Male parent	Col	69.8	1.00		
I	Bor-4	34.8	1.22	50.6	55.3 ± 1.3 (n = 7)
	Kin-0	9.3	1.19	36.9	36.9 ± 1.5 (n = 10)
	Pu2-23	21.1	1.07	44.6	46.9 ± 4.1 (n = 5)
	Rennes-11	15.2	0.75	46.4	43.5 ± 1.5 (n = 6)
	Sorbo	10.3	1.06	39.2	41.0 ± 2.2 (n = 5)
	Spr1-6	23.5	0.85	48.5	49.9 ± 2.6 (n = 6)
	Tamm-2	19.7	0.79	50.4	46.7 ± 3.8 (n = 5)
	Ts-5	22.1	1.04	45.5	41.8 ± 3.7 (n = 5)
	Zdr-1	9.3	0.89	41.3	37.8 ± 1.4 (n = 6)
	Zdr-6	19.9	1.43	40.4	43.4 ± 1.2 (n = 6)

II	Ga-0	26.1	0.72	51.5	40.6 ± 1.0 (n = 6)
	Sq-1	21.9	0.51	53.6	39.0 ± 1.1 (n = 5)

III	Bay-0	8.1	0.64	45.2	68.9 ± 0.9 (n = 4)
	CS22569	20.2	0.67	49.9	60.0 ± 1.1 (n = 5)
	KZ9	23.2	1.06	45.8	61.6 ± 0.9 (n = 5)
	Rennes-1	25.3	1.40	42.4	57.0 ± 1.4 (n = 6)

^a ^Average ± standard error (n, number of individuals).

**Figure 4:**
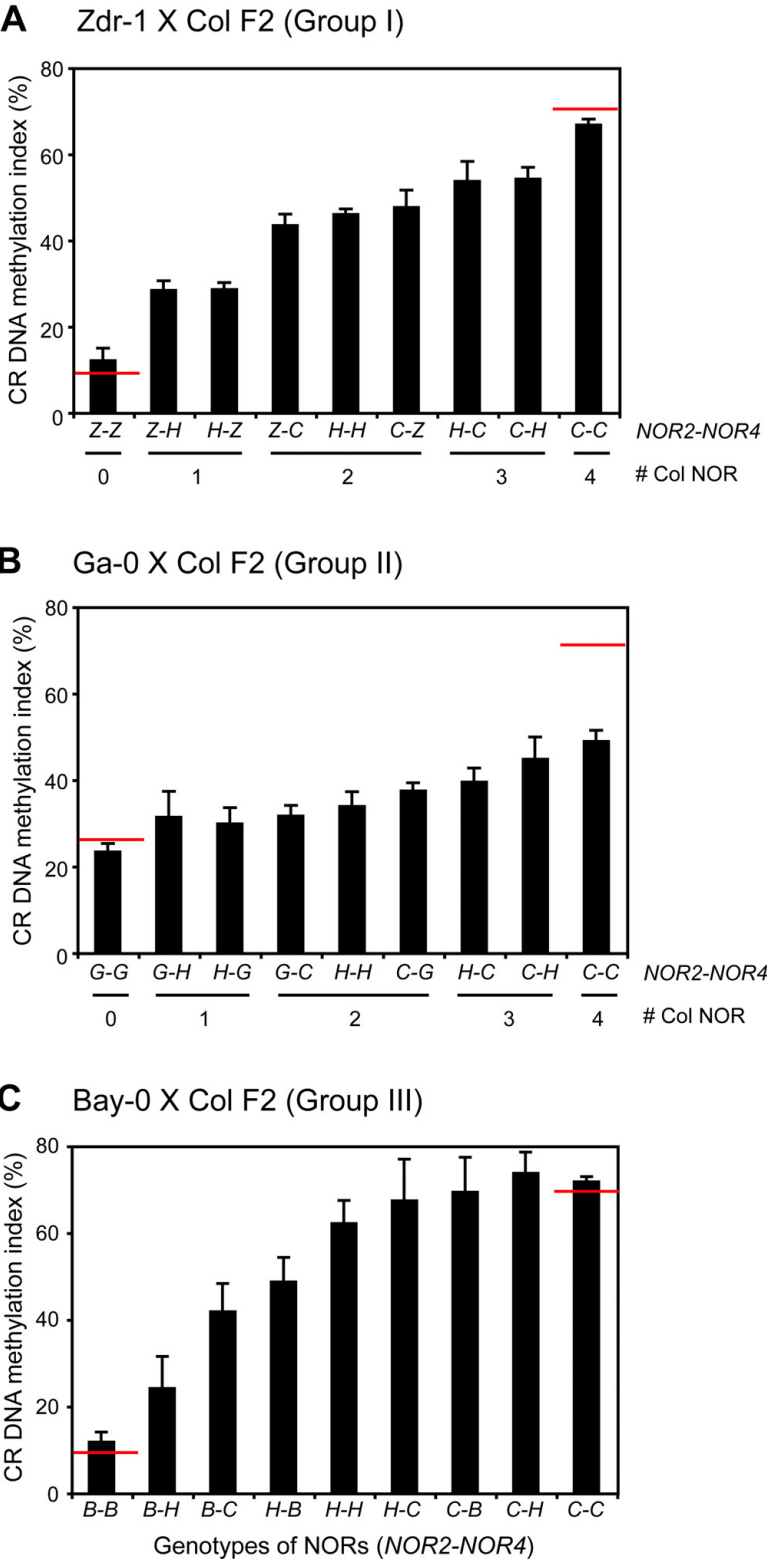
CR DNA methylation in F2 populations derived from crosses between Col and accessions with hypomethylated NORs. (A) Plot of CR DNA methylation index versus NOR genotype in a Zdr-1 × Col F2 population (group I). The F2 individuals were classified into nine groups based on their genotypes at *NOR2*-*NOR4 *(C = Col/Col, H = Col/Zdr-1, Z = Zdr-1/Zdr-1). (B) Plot of CR DNA methylation index versus NOR genotype in a Ga-0 × Col F2 population (group II) (C = Col/Col, H = Col/Ga-0, G = Ga-0/Ga-0). (C) Plot of CR DNA methylation index versus NOR genotype in a Bay-0 × Col F2 population (group III) (C = Col/Col, H = Col/Bay-0, B = Bay-0/Bay-0). Vertical bars denote standard errors. The values obtained for the parental accessions are indicated by red horizontal lines.

To further investigate the mechanisms controlling the inheritance of CR DNA methylation, we chose one F1 hybrid from each group and examined CR DNA methylation patterns in F2 populations derived by self-pollination of F1 hybrids. *NOR2 *and *NOR4 *are the main determinants for differential NOR DNA methylation in inter-strain crosses [26,30]; consequently, we analyzed whether the parental origin of each *NOR *affects total NOR DNA methylation in the F2 individuals. In a Zdr-1 × Col F2 population (group I), the range of CR DNA methylation content encompassed the values of both parents. A Zdr-1 × Col F2 population demonstrated the additive effect of *NOR2 *and *NOR4 *on overall NOR DNA methylation levels (Fig. 4A). The Ga-0 × Col F2 population (group II) showed that CR DNA methylation of F2 individuals was determined by the genotypes of each *NOR*, similar to the behavior seen in the group I cross, but the overall levels of CR DNA methylation were lower than those expected from a simple additive model of parental NOR DNA methylation inheritance (Fig. 4B). For example, individuals with a *NOR2-NOR4 *genotype of *C-C *(C = Col/Col) in the Ga-0 × Col F2 family showed a CR DNA methylation index of only ~50%, although the parental Col strain had a CR DNA methylation index of ~70%. In a Bay-0 × Col F2 population (group III), individuals where both copies of *NOR2 *were from the Bay-0 parent (genotypes for *NOR2-NOR4 *are *B-B*, *B-H*, and *B-C*, B = Bay-0/Bay-0, H = Col/Bay-0, and C = Col/Col) displayed a similar pattern of CR DNA methylation with F2 individuals from the group I cross (Zdr-1 × Col). However, individuals where both copies of *NOR2 *were from Col parent (*C-B*, *C-H*, and *C-C*) had a CR DNA methylation content similar to that of the Col parent, regardless of the origin of *NOR4 *(Fig. 4B). Our genetic analyses suggest that the inheritance of NOR DNA methylation in inter-strain crosses is regulated by multiple mechanisms, including faithful inheritance of parental NOR DNA methylation patterns and the reconfiguration of parental NOR DNA methylation in hybrids.

### NOR DNA methylation in the Bor-4 accession is determined by two different mechanisms

**Figure 5:**
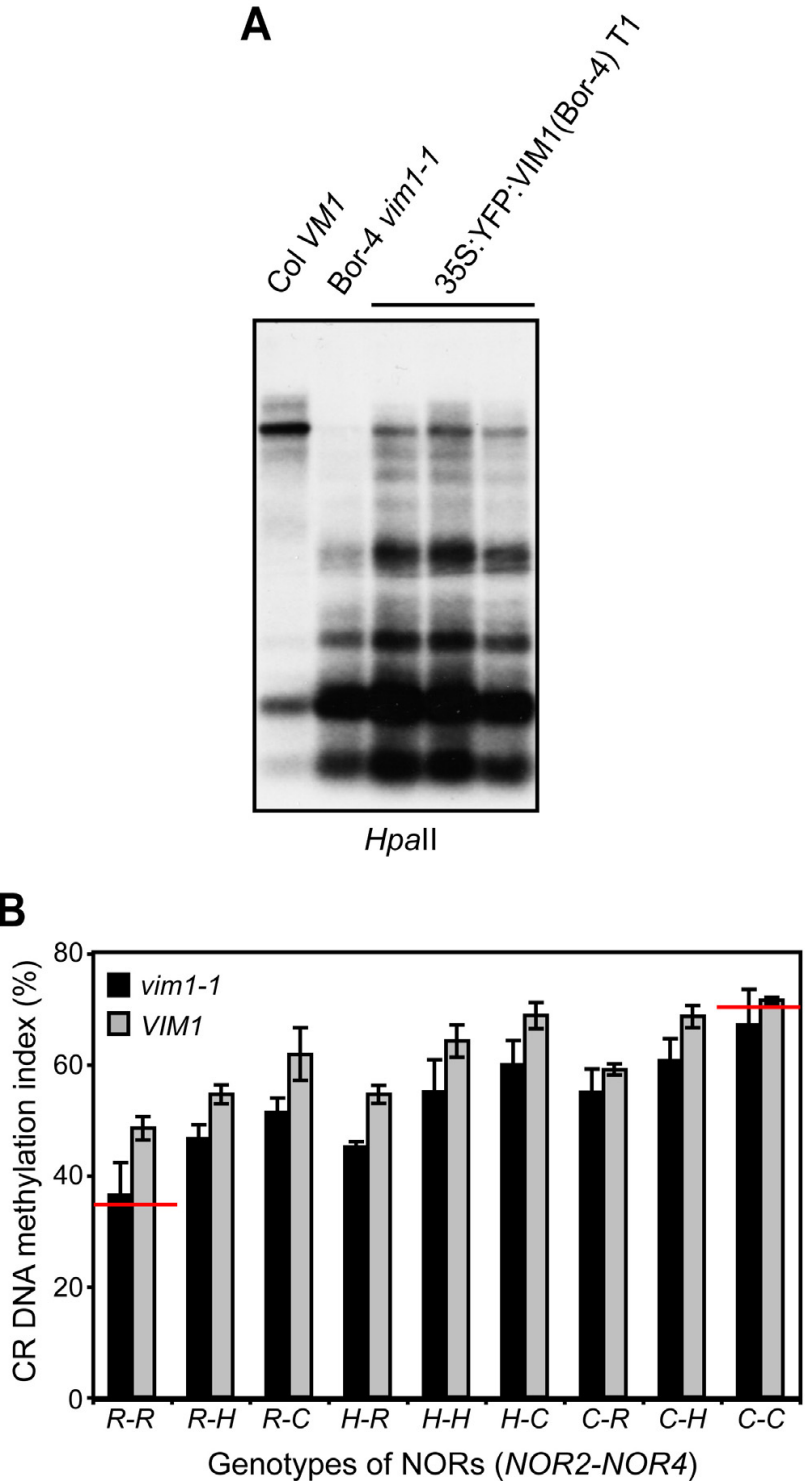
CR DNA methylation in the Bor-4 accession. (A) Increased NOR DNA methylation in Bor-4 plants transformed with a *VIM1 *transgene under the control of the 35S promoter. Genomic DNA samples of the indicated genotypes were digested with *Hpa*II and used for DNA gel blot analysis with a hybridization probe for the CR region. (B) Plot of CR DNA methylation index versus NOR and *VIM1 *genotypes in a Bor-4 × Col F2 population. The F2 individuals were classified into nine groups based on their genotypes at *NOR2*-*NOR4 *(C = Col/Col, H = Col/Bor-4, R = Bor-4/Bor-4), and then each group was divided into two groups based on the presence of the *vim1-1 *mutation as a homozygote. Vertical bars denote standard errors. The values obtained for the parental accessions are indicated by red horizontal lines.

Bor-4 contains the naturally-occurring null *vim1-1 *(*variant in methylation 1-1*) allele caused by a 3.2-kb deletion of *At1g57820 *(*VIM1*), which encodes an SRA domain methylcytosine-binding protein required for full methylation of the 180-bp centromere repeats [32]. Three other *vim1 *null alleles in the Col background also cause centromere hypomethylation, although the phenotypes in Col are weaker than those observed in Bor-4. The Col *vim1 *null mutants exhibit comparable DNA methylation to the standard wild-type Col strain in 45S rRNA gene repeats. However, it is still possible that the *vim1-1 *mutation in Bor-4 is a significant determinant of the relatively low CR DNA methylation (34.8 ± 0.5%) in this strain. To test this possibility we measured the CR DNA methylation in Bor-4 plants transformed with a transgene containing a functional *VIM1 *gene from strain Col. In a previous report we demonstrated that this transgene, which contains a Col *VIM1 *genomic fragment, successfully complements the centromere hypomethylation phenotype of Bor-4 [32]. In contrast, the CR DNA methylation index of Bor-4 plants with a Col *VIM1 *gene was increased, but not up to the level of the Col strain (38.6 ± 0.5%, n = 14) [see Additional File 5]. Next, we transformed Bor-4 plants with a transgene containing the Col *VIM1 *cDNA under the control of the strong, constitutive cauliflower mosaic virus 35S promoter. The CR DNA methylation index of *35S:YFP:VIM1 *(Bor-4) plants was 42.9 ± 0.4% (n = 6) (Fig. 5A), indicating that overexpression of a Col *VIM1 *cDNA in Bor-4 plants led to a slight increase of the CR DNA methylation. These observations suggest that restoration of *VIM1 *function in a Bor-4 background leads to a measurable increase in CR DNA methylation.

To weigh the contribution of *VIM1 *genotype on differential NOR DNA methylation in Bor-4, we examined CR DNA methylation in F1 hybrids between Bor-4 and Col, as well as F2 progeny. It is important to note that *vim1 *null mutations behave as recessive alleles relative to the functional wild-type Col *VIM1 *allele [32]. As shown in Table 1, Bor-4 belongs to group I: the average CR DNA methylation levels in Bor-4 × Col F1 individuals were intermediate between the two parental values. We measured CR DNA methylation content in Bor-4 × Col F2 individuals and determined the genotypes of each NOR. We found that CR DNA methylation levels were determined primarily by the additive effect of *NOR2 *and *NOR4 *(Fig. 5B), a pattern very similar to that observed in the group I Zdr-1 × Col F2 family shown in Figure 4A. Nonetheless, among individuals with the same genotypes for both NORs, individuals homozygous for the *vim1-1 *mutation had lower CR DNA methylation than those carrying a *VIM1 *allele (Fig. 5B). Our data demonstrate that CR DNA methylation in Bor-4 is mainly determined by the genotype or epigenotype at the NORs, but is modified by *trans*-acting genetic variation (i.e., the *vim1-1 *mutation).

## Discussion

In this study, we examined natural variation in NOR DNA methylation in 88 *A. thaliana *natural accessions. We extended our previous observations regarding the presence of significant natural variation in CR DNA methylation and the positive correlation between CR DNA methylation and rRNA gene copy number. Furthermore, our survey revealed natural variation in DNA methylation of the IGS region containing the transcriptional control elements of the 45S rRNA genes. The partial independence of CR and IGS DNA methylation suggests that independent regulatory mechanisms influence DNA methylation in two different regions of the 45S rRNA gene repeats. We also uncovered a range of mechanisms controlling the inheritance of NOR DNA methylation, including epigenetic inheritance of parental methylation patterns, reconfiguration of parental NOR DNA methylation in hybrids, and the involvement of *trans*-acting modifiers.

### Arabidopsis shows substantial natural variation in NOR DNA methylation

The molecular mechanisms involved in executing, regulating and interpreting epigenetic control of 45S rRNA gene transcription have been the subject of investigation in both animals [33-35] and plants [36,37]. In light of the growing body of evidence that DNA methylation plays an important role in epigenetic regulation of rRNA gene expression, our discovery of significant naturally-occurring variation in NOR DNA methylation in Arabidopsis is surprising. Our initial study of natural variation of NOR DNA methylation in Arabidopsis focused on a small number of accessions [26], but here we extended our investigation to 88 Arabidopsis natural accessions to evaluate the degree of natural variation in NOR DNA methylation (Fig. 1C, Additional File 2). While the upper range of CR methylation was similar to the values observed in our previous study, we found a number of accessions (Bay-0; 8.1 ± 0.8%, Kin-0; 9.3 ± 0.6%, and Zdr-1, 9.3 ± 0.2%) with extremely low CR DNA methylation indices that approached the index in Col *ddm1-2 *mutant plants (5.8 ± 0.9%). We note that the severe hypomethylation found in these strains is NOR-specific and other repetitive regions of the genome (e.g., 180-bp centromere repeats, 5S rRNA genes, *Athila *transposons) are methylated to a level similar to other accessions.

In addition to measuring total CR DNA methylation among Arabidopsis natural accessions, the combination of *Hpa*II and *Eco*RI digestion permitted a more detailed assessment of methylation patterns within the rRNA coding sequence. Specifically, *Hpa*II+*Eco*RI digestion provided a quantitative way to measure the portion of unmethylated (fraction a), partially methylated (fraction b), and fully methylated rRNA gene repeats (fraction c). The partitioning of hybridization signal among the three fractions defined general patterns, but some accessions deviated from the consensus patterns revealing that natural variation also exists for qualitative differences in DNA methylation distribution [see Additional File 4],

Our results indicate that rRNA gene copy number is a major predictor of NOR DNA methylation among Arabidopsis natural accessions (Fig. 2). Although the mechanistic basis of the association between these two parameters is not understood, excess rRNA genes might be transcriptionally silenced in association with increased DNA methylation. However, rRNA gene copy number does not fully account for all of the natural variation in NOR DNA methylation because strains with similar rRNA gene copy number can have dramatically different rRNA gene methylation levels; other factors must underlie the variation in CR DNA methylation.

### Inheritance of NOR DNA methylation

We investigated the inheritance of NOR DNA methylation by examining CR DNA methylation levels in F1 individuals generated by crosses between Col and 16 low CR DNA methylation accessions. The low CR DNA methylation accessions were divided into three different groups based on CR DNA methylation indices in F1 hybrids: intermediate between the two parental values (group I), versus significantly lower (group II) or higher (group III) CR DNA methylation than the weighted mid-parental value (Table 1). The prevalence of group I strains suggests that faithful epigenetic inheritance of parental DNA methylation is the norm, but reconfiguration of NOR DNA methylation patterns is not uncommon – represented by groups II and III. There are several explanations for reconfigured NOR DNA methylation in F1 hybrids of groups II and III, including interaction among the differentially methylated NORs. For example, the juxtaposition of a lightly methylated NOR with a more heavily methylated NOR could trigger loss or gain of DNA methylation from the rRNA gene arrays. Another possibility is that *trans*-acting modifiers act in a dominant fashion to reconfigure NOR methylation in the F1 generation. A third possibility is that the loss or gain of NOR DNA methylation in F1 hybrids is due to a change in copy number of rRNA genes in the hybrids, especially F1 plants resulting from group II crosses. It is noteworthy that the two accessions in group II have much smaller copy numbers of rRNA genes than the Col strain. The lower than expected levels of NOR DNA methylation in group II F1 hybrids (and the Ga-0 × Col F2 progeny) could be explained by the loss of heavily methylated rRNA gene repeats from the Col NORs. However, the nature of the triggers for reconfiguring parental NOR DNA methylation must be more complex considering that the Bay-0 × Col cross does not behave as predicted. In this case, bringing together small, lightly methylated NORs from Bay-0 with larger, heavily methylated NORs from Col results in more NOR DNA methylation in the F1 generation than expected.

We further investigated the mechanisms controlling the inheritance of CR DNA methylation in F2 populations. A Zdr-1 × Col F2 population demonstrated an additive effect of *NOR2 *and *NOR4 *on overall CR DNA methylation levels, consistent with the epigenetic inheritance of parental methylation patterns in group I accessions. The results of F2 segregation were more complex in the group II and group III crosses. For example, in the group III Bay-0 × Col F2 population, a major determinant of CR DNA methylation is the Col-derived *NOR2*, which influences DNA methylation on the Bay-0-derived *NOR4*. Our genetic analyses suggest that the epigenetic inheritance of NOR DNA methylation in inter-strain crosses is regulated by different mechanisms, including epigenetic inheritance of parental NOR DNA methylation patterns, crosstalk between NORs from different natural accessions, and/or the action of *trans*-acting modifiers. The relative contribution of these mechanisms differs dependent on which strains are hybridized.

### Mechanisms regulating NOR DNA methylation in Bor-4

Previous quantitative trait locus (QTL) analyses of NOR DNA methylation using Ler/Cvi recombinant inbred lines revealed three additional *trans*-acting QTLs located on chromosomes 1, 3, and 5, as well as two major QTLs which directly map on the target loci, *NOR2 *and *NOR4 *[26,30]. The identities of the genetic loci corresponding to these *trans*-acting QTLs are not known, but the *VIM1 *gene falls within the window defined by the QTL on chromosome 1. The Bor-4 accession contains the naturally-occurring *vim1-1 *loss-of-function allele; therefore, we used the Bor-4 strain to examine whether *VIM1 *could be a *trans*-acting modifier of NOR DNA methylation. While NOR DNA methylation in the Bor-4 × Col cross is determined primarily by the additive effects of the parental NORs, the transgenic and genetic segregation analyses shown in Figure 5 indicated that the *vim1-1 *mutation plays a significant role in specifying overall CR DNA methylation levels. This observation supports the possibility that *VIM1 *is responsible for the NOR DNA methylation QTL on chromosome 1.

## Conclusion

To address the genetic basis of NOR DNA methylation control and discern the factors that shape natural variation in NOR DNA methylation, we investigated the extent of natural variation in NOR DNA methylation in Arabidopsis natural accessions and the inheritance of parental NOR DNA methylation in inter-strain crosses. CR DNA methylation was positively correlated with copy number of 45S rRNA gene and IGS DNA methylation. Our results suggest that the NORs themselves are important determinants of natural variation in rRNA gene methylation and that the inheritance of NOR DNA methylation is controlled by a range of mechanisms, including epigenetic inheritance of parental methylation patterns, reconfiguration of parental NOR DNA methylation, and the involvement of *trans*-acting modifiers such as *VIM1*.

## Methods

### Plant materials

Seed for *A. thaliana *accessions collected by Nordborg *et al*. [27] were obtained from the Arabidopsis Biological Resource Center (ABRC) at The Ohio State University. The ABRC stock numbers are given in Additional File 2. Plants were grown in a growth chamber at 22°C under 16 hr of light, followed by 8 hr of darkness.

### DNA gel blot analysis

Leaf tissues from multiple individual plants were collected for preparing genomic DNA from at least three biological replications. Genomic DNA was digested with *Hpa*II or *Hpa*II+*Eco*RI according to the manufacturer's (New England Biolabs, USA) instructions. Radiolabeled probes were generated by random priming, and blots were prepared and hybridized using standard methods. The following hybridization probes were generated from purified cloned inserts: a 3.7 kb *Eco*RI fragment containing the 5.8S and 25S rRNA gene from plasmid pARR17 [28] and a 1.7 kb *Eco*RI fragment from IGS clone, pAt4 [26,37]. CR and IGS DNA methylation levels were determined from phosphorimager (Bio-Rad, USA) files using Quantity One™ (Bio-Rad, USA) software.

### rRNA gene copy number

45S rRNA gene copy number was determined by DNA gel blot analysis. Genomic DNA from each accession was digested with *Eco*RI (New England Biolabs, USA). The blots were hybridized with a single-copy probe containing *DDM1 *and subsequently hybridized with the CR probe. The hybridization signals were quantified using phosphorimager analysis (Bio-Rad, USA), and the ratios between the rRNA genes signal and single-copy signal were calculated and then normalized to the ratio for Col.

### F1 and F2 analysis

Since the direction of the cross does not have a significant effect on CR DNA methylation (data not shown; [26]), we used Col as a paternal parent and the low CR methylation accessions as maternal parents for the inter-strain crosses. At least four F1 individuals for each cross and three F2 individuals per each *NOR2*-*NOR4 *genotype combination were assayed for the CR DNA methylation indices using DNA gel blot analysis, as described in the previous section. F2 individuals were genotyped for one marker close to *NOR2*, *F10A8-12*, and one marker close to *NOR4*, *F6N15-34*. For the Bor-4 × Col F2 analysis, F2 individuals were also genotyped for *VIM1 *using VIM1-A and VIM1-B primers. Primer sequences for these markers are listed in Additional File 6.

### Construction of plant expression vectors and generation of transgenic plants

The transgenic lines used in this study were constructed as previously described [32]. The 5.5-kb Col genomic DNA fragment containing the whole predicted ORF of *VIM1 *with 2.0 kb of the promoter region and a full-length Col *VIM1 *cDNA clone were cloned into pENTR-D TOPO (Invitrogen, USA), and the resulting *VIM *inserts were recombined into pEarlyGate302 (for the genomic fragment) and pEarlyGate104 (for the cDNA fragment) [38] using Gateway technology (Invitrogen, USA). These constructs were transformed into *Agrobacterium tumefaciens *(LBA4404) and were introduced into Bor-4 *vim1-1 *plants by *in planta *transformation [39].

## Abbreviations

NOR: nucleolus organizer region; rRNA: ribosomal RNA; CR: coding region of the 5.8S and 25S rRNA genes; IGS: intergenic spacer region of the rRNA genes; QTL: quantitative trait locus.

## Authors' contributions

HRW designed the studies, carried out all of the experiments, analyzed the data and wrote the manuscript. EJR participated in the design of the study, analysis of the data, and the writing of the manuscript. Both authors read and approved the final manuscript.

## Supplementary Material

Additional file 1 Genomic DNA blot analysis of CR DNA methylation among different natural accessions of Arabidopsis. See Figure 1B for details. Click here for file

Additional file 2 CR DNA methylation index and relative rRNA gene copy number in different natural accessions of Arabidopsis. Click here for file

Additional file 3 CR DNA methylation after *Hpa*II+*Eco*RI digestion among Arabidopsis natural accessions. (A) A representative genomic DNA blot analysis of Arabidopsis natural accessions using the CR probe after simultaneous digestion of *Hpa*II and *Eco*RI. Fraction a, unmethylated fragments; fraction b, partially methylated fragments; fraction c, fully methylated fragments. (B) Correlation between CR DNA methylation indices based on *Hpa*II versus *Hpa*II+*Eco*RI digestion. The line is a linear regression of the data (linear regression coefficient, R = 0.99). Click here for file

Additional file 4 Detailed CR DNA methylation patterns of natural accessions with hypermethylated or hypomethylated NORs. (A-E) Plots of CR DNA methylation hybridization intensity in three fractions described in Additional File 2A. (A) A common CR DNA methylation pattern in hypermethylated accessions (>65% CR DNA methylation index). (B) Two exceptional accessions with high CR methylation indices. (C) A common CR DNA methylation pattern in hypomethylated accessions (<30% CR DNA methylation index). (D) and (E) Qualitatively different DNA methylation patterns in natural accessions with hypomethylated NORs (in these cases, ~30% CR DNA methylation index). The relative hybridization signal intensity from unmethylated fragments (fraction a), partially methylated fragments (fraction b), and the fully methylated, undigested 3.7 kb fragments (fraction c) were measured from each natural accession. Click here for file

Additional file 5 Increased NOR DNA methylation in Bor-4 plants transformed with a fragment containing the genomic *VIM1 *gene from strain Col. Genomic DNA samples of the indicated genotypes were digested with *Hpa*II and used for DNA gel blot analysis with a hybridization probe for the CR region. The slight increase in CR DNA methylation can be detected visually by comparing the intensity of the uppermost bands relative to darkest band in the top half of the blot. Click here for file

Additional file 6 Oligonucleotide primers used in this study. Click here for file
