# Supplementary figures and images for: Natural variation in DNA methylation in ribosomal RNA genes of Arabidopsis thaliana
Source: BMC Plant Biol. 2008 Sep 10;8:92. doi: 10.1186/1471-2229-8-92 (PMC2551617; doi:10.1186/1471-2229-8-92)

Additional File 1

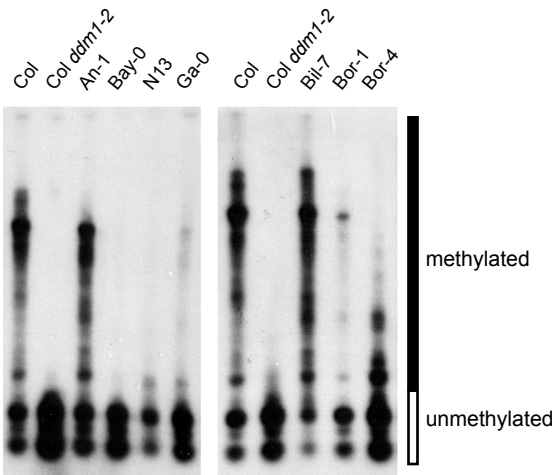

Supplement: Additional file 1 — Genomic DNA blot analysis of CR DNA methylation among different natural accessions of Arabidopsis. See Figure 1B for details. [file 1471-2229-8-92-S1.pdf]

# Additional File 3

**A**

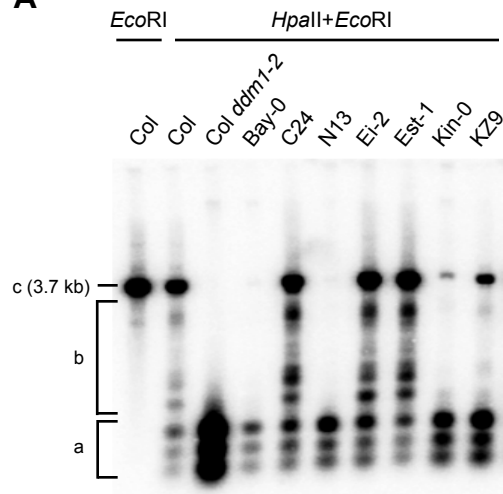

**B**

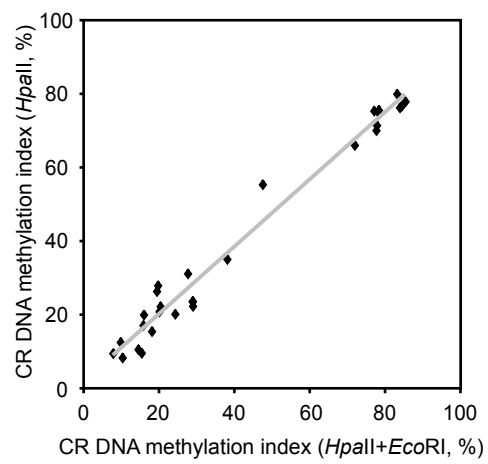

Supplement: Additional file 3 — CR DNA methylation after HpaII+EcoRI digestion among Arabidopsis natural accessions. (A) A representative genomic DNA blot analysis of Arabidopsis natural accessions using the CR probe after simultaneous digestion of HpaII and EcoRI. Fraction a, unmethylated fragments; fraction b, partially methylated fragments; fraction c, fully methylated fragments. (B) Correlation between CR DNA methylation indices based on HpaII versus HpaII+EcoRI digestion. The line is a linear regression of the data (linear regression coefficient, R = 0.99). [file 1471-2229-8-92-S3.pdf]

# Additional File 4

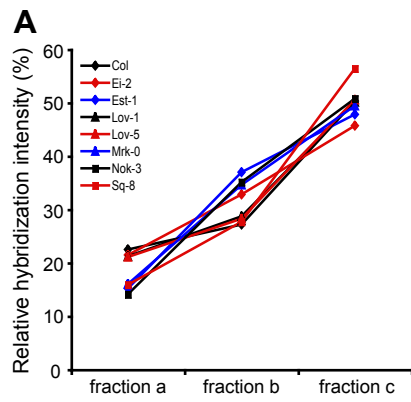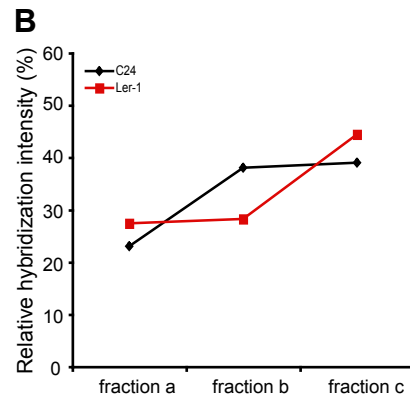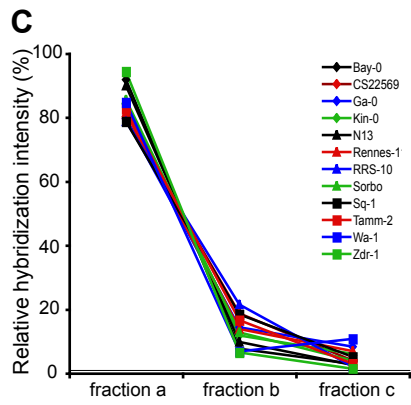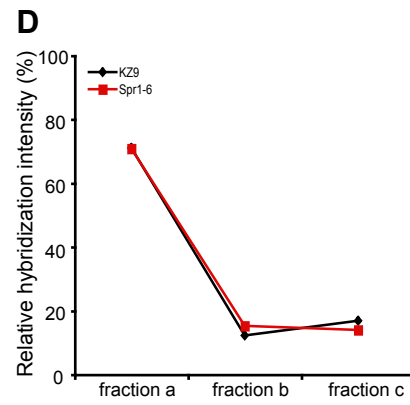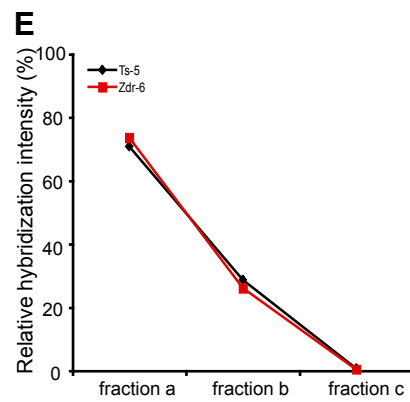

Supplement: Additional file 4 — Detailed CR DNA methylation patterns of natural accessions with hypermethylated or hypomethylated NORs. (A-E) Plots of CR DNA methylation hybridization intensity in three fractions described in Additional File 2A. (A) A common CR DNA methylation pattern in hypermethylated accessions (>65% CR DNA methylation index). (B) Two exceptional accessions with high CR methylation indices. (C) A common CR DNA methylation pattern in hypomethylated accessions (<30% CR DNA methylation index). (D) and (E) Qualitatively different DNA methylation patterns in natural accessions with hypomethylated NORs (in these cases, ~30% CR DNA methylation index). The relative hybridization signal intensity from unmethylated fragments (fraction a), partially methylated fragments (fraction b), and the fully methylated, undigested 3.7 kb fragments (fraction c) were measured from each natural accession. [file 1471-2229-8-92-S4.pdf]

Additional File 5

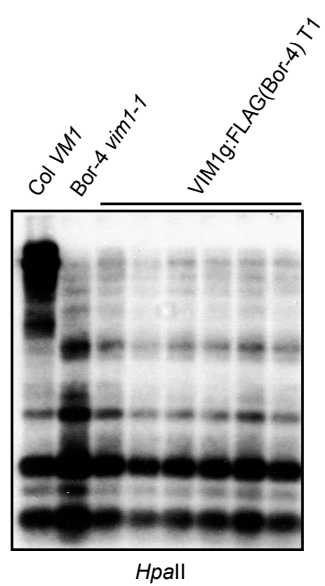

Supplement: Additional file 5 — Increased NOR DNA methylation in Bor-4 plants transformed with a fragment containing the genomic VIM1 gene from strain Col. Genomic DNA samples of the indicated genotypes were digested with HpaII and used for DNA gel blot analysis with a hybridization probe for the CR region. The slight increase in CR DNA methylation can be detected visually by comparing the intensity of the uppermost bands relative to darkest band in the top half of the blot. [file 1471-2229-8-92-S5.pdf]
