# Supplementary material for: Natural variation in DNA methylation in ribosomal RNA genes of Arabidopsis thaliana
Source: BMC Plant Biol. 2008 Sep 10;8:92. doi: 10.1186/1471-2229-8-92 (PMC2551617; doi:10.1186/1471-2229-8-92)
Supplement: Additional file 2 — CR DNA methylation index and relative rRNA gene copy number in different natural accessions of Arabidopsis. [file 1471-2229-8-92-S2.pdf]

Additional File 2. CR DNA methylation index and relative rRNA gene copy number in different natural accessions of Arabidopsis

Accessions	ABRC stock no.	CR DNA methylation index (%)[a]	IGS DNA methylation index (%)[a]	Relative rRNA gene copy number[a]
Ag-0	CS22630	54.8 ± 0.4	52.0 ± 0.7	
An-1	CS22626	59.5 ± 0.2	32.8 ± 2.3	
Bay-0	CS22633	8.1 ± 0.8	38.9 ± 0.5	0.64 ± 0.05
Bil-7	CS22579	82.5 ± 0.6	69.1 ± 1.4	1.23 ± 0.04
Bor-1	CS22590	46.6 ± 2.8	29.8 ± 1.9	0.73 ± 0.08
Bor-4	CS22591	34.8 ± 0.5	35.2 ± 1.3	1.22 ± 0.04
Br-0	CS22628	39.7 ± 2.0		
Bur-0	CS22656	49.0 ± 0.5	61.7 ± 0.1	
C24	CS22620	75.1 ± 0.1	39.1 ± 1.8	1.13 ± 0.10
CIBC5	CS22602	60.7 ± 1.0		
CIBC17	CS22603	50.5 ± 2.9	39.1 ± 1.0	
Col	CS22625	69.8 ± 0.6	73.3 ± 1.2	1.00 ± 0.00
CS22568	CS22568	67.6 ± 1.4	32.7 ± 2.4	
CS22569	CS22569	20.2 ± 2.5	29.6 ± 1.0	0.67 ± 0.08
Ct-1	CS22639	40.9 ± 0.3	27.9 ± 0.9	1.13 ± 0.01
Cvi-0	CS22614	30.9 ± 1.3	25.5 ± 0.8	0.91 ± 0.05
Eden-1	CS22572	38.2 ± 1.9	45.9 ± 0.1	
Edi-0	CS22657	38.2 ± 2.8	37.9 ± 2.9	0.83 ± 0.08
Ei-2	CS22616	71.2 ± 0.7	55.9 ± 2.8	1.69 ± 0.05
Est-1	CS22629	76.5 ± 1.0	56.4 ± 2.7	1.37 ± 0.12
Fei-0	CS22645	56.9 ± 1.5		
Ga-0	CS22634	26.1 ± 0.6	40.7 ± 1.9	0.72 ± 0.14
Goettingen-7	CS22608	69.9 ± 2.3	47.2 ± 2.2	
Goettingen-22	CS22609	66.3 ± 2.5	33.2 ± 0.1	
Gu-0	CS22617	32.1 ± 1.6	19.5 ± 1.6	
Gy-0	CS22631	46.0± 0.8		
HR-5	CS22596	47.7 ± 0.2	33.9 ± 0.1	0.83 ± 0.05
HR-10	CS22597	42.2 ± 1.9	35.7 ± 0.3	
Kas-1	CS22638	55.7 ± 0.1		1.13 ± 0.07
Kin-0	CS22654	9.3 ± 0.6	20.9 ± 3.2	1.19 ± 0.11
Knox-10	CS22566	49.7 ± 0.6	31.5 ± 1.8	
Knox-18	CS22567	55.6 ± 2.1	58.4 ± 2.4	
Kondara	CS22651	70.7 ± 1.7		
KZ1	CS22606	72.4 ± 1.5	92.3 ± 0.1	2.05 ± 0.17
KZ9	CS22607	23.2 ± 3.0	22.1 ± 0.1	1.06 ± 0.04
Ler-1	CS22618	65.8 ± 1.3	76.8 ± 0.1	0.93 ± 0.06
Ll-0	CS22650	50.0 ± 1.3	34.2 ± 2.8	

Lov-1	CS22574	74.5 ± 0.4	41.2 ± 2.4	1.62 ± 0.11
Lov-5	CS22575	75.3 ± 0.1	48.0 ± 0.8	1.46 ± 0.18
Lp2-2	CS22594	29.7 ± 1.6	38.3 ± 0.1	0.56 ± 0.07
Lp2-6	CS22595	36.0 ± 0.7		
Lz-0	CS22615	38.8 ± 2.3	28. 9 ± 0.6	
Mrk-0	CS22635	79.7 ± 0.7	67.0 ± 2.9	1.27 ± 0.09
Ms-0	CS22655	33.6 ± 2.0	42.9 ± 1.7	
Mt-0	CS22642	47.4 ± 0.2	29.6 ± 1.3	
Mz-0	CS22636	46.7 ± 0.1	37.1 ± 1.6	
N13	CS22621	12.2 ± 1.1	28.7 ± 3.2	0.69 ± 0.13
Nd-1	CS22619	65.5 ± 0.6	75.1 ± 1.8	
NFA-8	CS22598	47.1 ± 1.8	41.8 ± 3.4	
NFA-10	CS22599	48.4 ± 1.1		
Nok-3	CS22643	77.7 ± 1.1	60.0 ± 5.3	1.89 ± 0.11
Omo2-1	CS22584	49.4 ± 2.4	28.7 ± 2.8	
Omo2-3	CS22585	36.8 ± 2.3	28.8 ± 0.8	
Oy-0	CS22658	63.8 ± 1.7	66.6 ± 2.8	
Pna-10	CS22571	41.9 ± 4.7	27.5 ± 3.4	0.69 ± 0.10
Pna-17	CS22570	50.5 ± 0.8	39.8 ± 0.1	
Pro-0	CS22649	49.7 ± 2.4	30.9 ± 3.5	
Pu2-23	CS22593	21.1 ± 1.6	28.1 ± 2.5	1.07 ± 0.03
Pu2-7	CS22592	62.3 ± 1.7	47.8 ± 3.4	
Ra-0	CS22632	43.3 ± 0.6	28.6 ± 0.1	
Rennes-1	CS22610	25.3 ± 2.3	38.9 ± 4.7	1.40 ± 0.14
Rennes-11	CS22611	15.2 ± 1.4	35.1 ± 1.2	0.75 ± 0.06
RRS-7	CS22564	30.7 ± 0.6	30.2 ± 0.1	
RRS-10	CS22565	27.6 ± 0.5	34.5 ± 1.9	0.75 ± 0.06
Se-0	CS22646	33.1 ± 0.5		
Shahdara	CS22652	53.9 ± 2.1	39.8 ± 1.0	
Sorbo	CS22653	10.3 ± 0.5		1.06 ± 0.08
Spr1-2	CS22582	66.1 ± 1.8	51.1 ± 2.5	
Spr1-6	CS22583	23.5 ± 2.4	23.3 ± 0.2	0.85 ± 0.11
Sq-1	CS22600	21.9 ± 2.1	29.0 ± 5.1	0.51 ± 0.01
Sq-8	CS22601	76.0 ± 0.1	76.1 ± 0.1	1.67 ± 0.08
Tamm-2	CS22604	19.7 ± 1.1	27.9 ± 2.2	0.79 ± 0.11
Tamm-27	CS22605	30.3 ± 0.1	27.4 ± 0.9	0.77 ± 0.04
Ts-1	CS22647	64.9 ± 0.4	31.4 ± 2.9	
Ts-5	CS22648	22.1 ± 1.2	32.2 ± 0.1	1.04 ± 0.13
Tsu-1	CS22641	46.4 ± 1.0	34.8 ± 2.3	
Ull2-3	CS22587	46.7 ± 0.5	38.8 ± 0.7	
Uod-1	CS22612	56.9 ± 1.5	36.9 ± 5.8	
Uod-7	CS22613	44.3 ± 4.5	35.4 ± 2.1	
Van-0	CS22627	60.0 ± 2.2		
Wa-1	CS22644	16.9 ± 1.8	23.5 ± 2.3	0.68 ± 0.10
Wei-0	CS22622	45.4 ± 0.4		

Ws-0	CS22623	55.5 ± 1.1		
Ws-2	CS22659	55.1 ± 2.2	42.1 ± 1.4	0.86 ± 0.03
Wt-5	CS22637	75.2 ± 3.5	84.2 ± 1.6	
Yo-0	CS22624	66.4 ± 1.2	38.2 ± 1.8	
Zdr-1	CS22588	9.3 ± 0.2	29.0 ± 3.1	0.89 ± 0.05
Zdr-6	CS22589	19.9 ± 0.3	32.3 ± 1.0	1.43 ± 0.07

[a] Average ± standard error.
