# Supplementary material for: Natural variation in DNA methylation in ribosomal RNA genes of Arabidopsis thaliana
Source: BMC Plant Biol. 2008 Sep 10;8:92. doi: 10.1186/1471-2229-8-92 (PMC2551617; doi:10.1186/1471-2229-8-92)
Supplement: Additional file 6 — Oligonucleotide primers used in this study. [file 1471-2229-8-92-S6.pdf]

Additional File 6. Oligonucleotide primers used in this study

Name	Sequence (5′→3′)	Description
F10A8-12F	ccacgcagcttccttcacagcca	Genotype for *NOR2*; Generates an amplicon of varying size depending on the strain background.
F10A8-12R	gtcatatatttcaccatgttatc	
F6N15-34F	ctaagcagttgttgtgtttcact	Genotype for *NOR4*; Generates an amplicon of varying size depending on the strain background.
F6N15-34R	ctccttctagaaacagaacacca	
VIM1-A	atggcgcgtgacatccaactcccc	Genotype for *VIM1;* Generates an amplicon of varying size for the vim1-1 deletion allele relative to Col VIM1.
VIM1-B	ggtatcaggaaactaacctgctg	
